# Alternative Splicing within and between *Drosophila* Species, Sexes, Tissues, and Developmental Stages

**DOI:** 10.1371/journal.pgen.1006464

**Published:** 2016-12-09

**Authors:** Lauren Gibilisco, Qi Zhou, Shivani Mahajan, Doris Bachtrog

**Affiliations:** Department of Integrative Biology, University of California, Berkeley, Berkeley, CA, United States of America; Stanford University School of Medicine, UNITED STATES

## Abstract

Alternative pre-mRNA splicing (“AS”) greatly expands proteome diversity, but little is known about the evolutionary landscape of AS in *Drosophila* and how it differs between embryonic and adult stages or males and females. Here we study the transcriptomes from several tissues and developmental stages in males and females from four species across the *Drosophila* genus. We find that 20–37% of multi-exon genes are alternatively spliced. While males generally express a larger number of genes, AS is more prevalent in females, suggesting that the sexes adopt different expression strategies for their specialized function. While the number of total genes expressed increases during early embryonic development, the proportion of expressed genes that are alternatively spliced is highest in the very early embryo, before the onset of zygotic transcription. This indicates that females deposit a diversity of isoforms into the egg, consistent with abundant AS found in ovary. Cluster analysis by gene expression (“GE”) levels shows mostly stage-specific clustering in embryonic samples, and tissue-specific clustering in adult tissues. Clustering embryonic stages and adult tissues based on AS profiles results in stronger species-specific clustering, suggesting that diversification of splicing contributes to lineage-specific evolution in *Drosophila*. Most sex-biased AS found in flies is due to AS in gonads, with little sex-specific splicing in somatic tissues.

## Author Summary

Alternative pre-mRNA splicing (“AS”) greatly expands the proteome diversity within and between species by creating different combinations of exons from the same genomic loci. Recent comparisons of transcriptomes of equivalent adult organs in several vertebrate species revealed that AS differs significantly in its complexity across tetrapods, and may contribute to lineage-specific adaptation in vertebrates. AS is also prevalent in *Drosophila*, and has been most extensively studied in *D*. *melanogaster*. However, the spatial and temporal evolution of AS between *Drosophila* species remains to be explored. Here we study the transcriptomes from several tissues and developmental stages in males and females from four *Drosophila* species, spanning a major phylogenetic range of *Drosophila*. We show that while males generally express a larger number of genes, AS is more prevalent in females. This suggests that the sexes adopt different expression strategies for their specialized function–i.e. males tend to employ different genes, while females tend to utilize different isoforms. We also find that the dynamics of AS change over early development; while the number of total genes expressed increases during early embryonic development, the proportion of expressed genes that are alternatively spliced is highest in the very early embryo, before the onset of zygotic transcription. This indicates that females deposit a diversity of isoforms into the egg, consistent with abundant AS found in ovary. Like in mammals, cluster analysis suggests that diversification of splicing also significantly contributes to lineage-specific adaptation within the *Drosophila* genus.

## Introduction

Alternative pre-mRNA splicing (“AS”) greatly expands the proteome diversity within species by creating different combinations of exons from the same genomic loci [1]. The resulting mRNA isoforms are usually expressed in a tissue or developmental-stage specific manner and underlie numerous essential biological processes like sex-determination [2, 3], tissue development [4], and stress response [5]. AS can also greatly increase the proteome diversity between species with similar repertoires of protein-coding genes [6, 7]. Given the correlation of alternative splicing with the evolution of organismal complexity [1], its dynamics across developmental stages, tissues, and species have attracted great attention [6, 7]. Recent comparisons of transcriptomes of equivalent adult organs in several vertebrate species revealed that AS differs significantly in its complexity across the studied tetrapods, with primates showing the highest abundance of alternative splicing events [6, 7]. Interestingly, AS shows a greater level of interspecific divergence and lineage-specific turnover across tissues than absolute gene expression levels, suggesting that the diversification of splicing significantly contributes to lineage-specific adaptation [6, 7].

AS is also prevalent in *Drosophila*, and has been most extensively studied in *D*. *melanogaster* [8–16]. Over half of the spliced *D*. *melanogaster* genes encode two or more transcript isoforms, with about 50 genes capable of encoding over 1000 transcript isoforms each [17]. The complexity of AS also differs across developmental stages and tissues [18, 19] and different environmental perturbations [17]. However, the spatial and temporal evolution of AS between *Drosophila* species remains to be explored. Here we study the transcriptomes from several tissues and developmental stages in males and females from four *Drosophila* species, spanning a major phylogenetic range of *Drosophila*. In particular, we analyze two species pairs that diverged from each other at varying evolutionary distances: *D*. *pseudoobscura* and its sister species *D*. *miranda* split about 2 million years (“MY”) ago [20], and they diverged from *D*. *melanogaster* roughly 25 MY ago [21]; *D*. *nasuta* and *D*. *albomicans* split only about 0.1 MY ago [22], and diverged from *D*. *melanogaster* over 60 MY ago [23]. The presence of genomic resources for these species [24–27] combined with their varying split times allows us to study the evolution of AS on different time scales in different lineages, and enables us to address novel aspects of transcriptome diversity, such as the evolutionary landscape of AS in *Drosophila*, and how it differs between embryonic and adult stages and males and females.

## Results

### Transcriptome diversity across species

**Fig 1:**
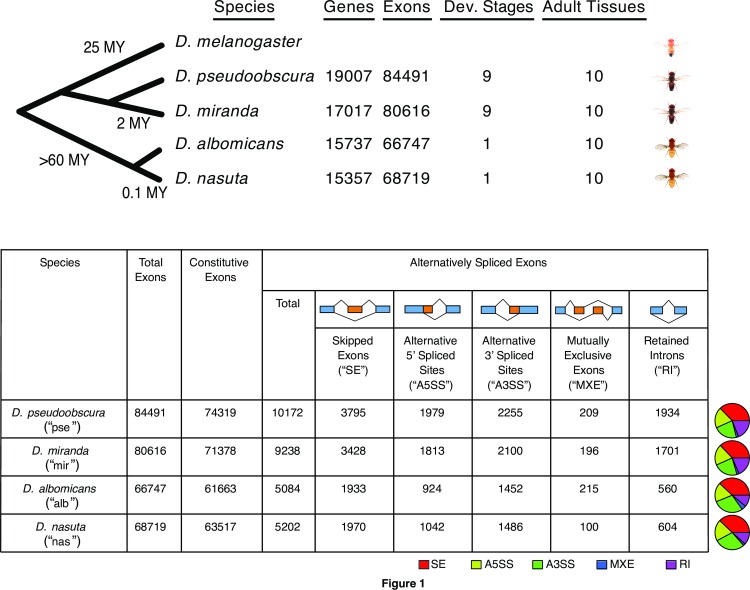
Overview of RNA-seq data used for analyses and alternative splicing in four *Drosophila* species. Top: Numbers of genes and exons detected and numbers of stages/tissues for each of four *Drosophila* species. Bottom: Numbers of total, constitutive, and alternatively spliced exons, as well as a breakdown of types of alternatively spliced exons, for each species. Proportions of different types of alternatively spliced exons are shown as pie charts to the right of the table.

We used RNA-seq data for different tissues in *D*. *pseudoobscura*, *D*. *miranda*, *D*. *albomicans*, and *D*. *nasuta* from males and females (one female and one male larval stage and five female and five male adult samples; **S1 Table**). In addition, we used published data derived from sexed early embryonic stages (eight female and eight male embryonic stages) in *D*. *pseudoobscura* and *D*. *miranda* spanning the onset of zygotic transcription [24]. We analyzed a total of 144 samples from different tissues or developmental stages, summing up to a total of 6,453,796,999 reads. An overview of the data used for each species is displayed in **Fig 1**.

The large number of stages and tissues allowed us to comprehensively annotate the transcriptomes of the four species and a summary of the gene annotations and alternative splicing events is outlined in **Fig 1**. We considered skipped exons, alternative 5’ and 3’ spliced sites, mutually exclusive exons, and retained introns. We annotated between 15,357 and 19,007 genes, and between 66,747 and 84,491 exons for each species (**Fig 1**). We find that between 20 and 37% of all multi-exon genes are alternatively spliced in at least one tissue or stage and between 5,084 and 10,172 exons (8–12% of all exons) are alternatively spliced (**Fig 1**). These values are similar to reports in *D*. *melanogaster* (where 31% of genes were found to be alternatively spliced; [14]), but considerably lower than the degree of AS in mammals, where almost 100% of multi-exon genes are alternatively spliced [7]. Less AS in *Drosophila* is consistent with findings that species further from primates have lower proportions of exons undergoing alternative splicing [6].

As expected, we detected more genes, exons, and AS events for the two species (*D*. *pseudoobscura* and *D*. *miranda*) for which we had both more comprehensive sampling (more samples and RNA-seq reads) and higher quality genome assemblies. Thus, while differences in the numbers of genomic features detected may in part reflect real species differences, less power to annotate genes, exons, and AS events in *D*. *albomicans* and *D*. *nasuta* probably largely contributes to these differences. Note that genomic features shown in **Fig 1** represent our within-species annotations (see Methods), and differences in these numbers between species should not greatly affect our comparisons of different tissues/samples within single species. For interspecific comparisons, we focused our analysis on a subset of expressed genes and exons for which we could identify orthologs from all four species (see Methods).

Skipped exons are the most abundant alternative splicing event in all four species, and mutually exclusive exons are the least abundant, consistent with previous studies in *D*. *melanogaster* [14, 28]. The relative percentage of each type of AS is generally similar between the studied *Drosophila* species (**Fig 1**), despite their evolutionary distance or different numbers of tissues and developmental stages sampled, indicating a high level of conservation of AS composition.

### Temporal and spatial expression dynamics within species

**Fig 2:**
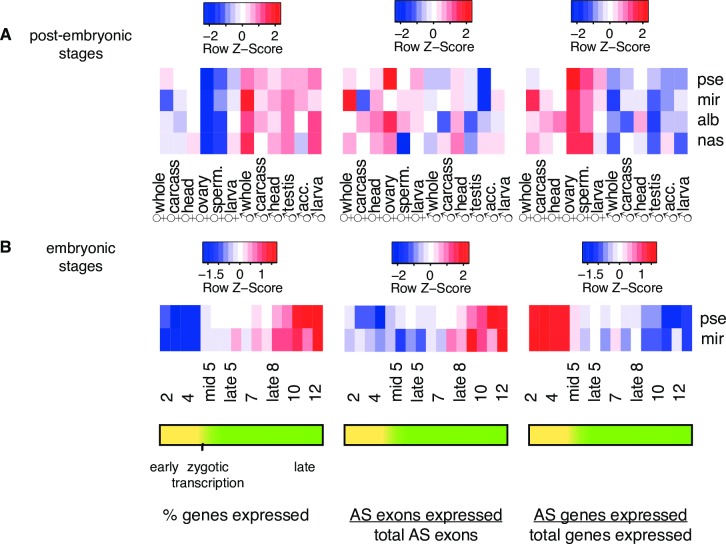
Gene expression and alternative splicing profiles across tissues and development. Comparisons of % of total genes expressed (left panel), % annotated alternatively spliced exons expressed (middle panel), and % of total genes expressed that are annotated as alternatively spliced (right panel) for **(A)** post-embryonic tissues and **(B)** embryonic stages. Each row of each heatmap is scaled separately by Z-score. *“*pse” = *D*. *pseudoobscura*; “mir” = *D*. *miranda*; “alb” = *D*. *albomicans*; “nas” = *D*. *nasuta*; “sperm.” = spermatheca; “larva” = 3^rd^ instar larva; “acc.” = accessory gland

We examined the transcriptome composition across corresponding samples of the four species using both the abundance of expressed genes and abundance of different alternatively spliced exons. Consistent with previous findings [29], males generally express more genes than females across almost all tissues (**Fig 2A**, left panel). For example, 63–80% of all genes are expressed in male whole body while only 47–64% of all genes are expressed in female whole body. The largest discrepancy in the number of expressed genes between sexes is found in adult gonads, with testis expressing 1.6–1.7x more genes than ovary (58–76% of all genes are expressed in testis while only 35–47% of all genes are expressed in ovary). That, however, does not necessarily mean ovary or female tissues have lower transcriptome diversity. While female samples usually show fewer expressed genes, the fraction of expressed genes that are alternatively spliced is generally higher in females than in males (**Fig 2A**, right panel). Ovary and spermatheca have the highest percentage of expressed genes annotated as alternatively spliced (24–45% in ovary and 24–40% in spermatheca; **Fig 2A**, right panel) despite showing among the lowest percentage of expressed genes. This indicates that male and female reproductive tissues may adopt different expression strategies for their specialized function: male tissues increase their transcriptome diversity by expressing different types of genes, while female tissues rely more on AS to increase the number of transcripts.

Across the four species, between 54–71% of all genes are expressed in head, and head generally shows relatively high proportions of all annotated alternative exons expressed (8–13%; **Fig 2A**, middle panel). This is consistent with earlier studies in *Drosophila* and humans, which show high transcriptional diversity in brain [30, 31]. Note that while the general trends reported above hold for all four species, exact proportions of genes and exons expressed and alternatively spliced vary between specific tissues and sexes. Differences in genome assembly quality and sampling among the four species, as well as species-specific idiosyncrasies, probably contribute to these differences.

In addition, we also analyzed developmental time course data in *D*. *miranda* and *D*. *pseudoobscura*, focusing on early embryonic development. The analyzed data encompass the maternal to zygotic transition (which happens during stage 5, see **Fig 2B**), when maternal transcripts begin to degrade and widespread zygotic transcription is initiated [24]. This allows us to contrast the landscape of alternative splicing between genes contributed maternally with those transcribed in the early embryo. Over development, both the percentage of total genes expressed (**Fig 2B**, left panel) and the percentage of alternatively spliced exons expressed (**Fig 2B**, middle panel) increase as development proceeds in both males and females. However, the proportion of expressed genes that are alternatively spliced is the highest in early embryonic development, before the onset of zygotic transcription, and drops in later stages as single-transcript gene expression increases (**Fig 2B**, right panel). This indicates that the mother deposits a diversity of isoforms into the egg and that early zygotic transcription increases the number of genes expressed, but most of those genes do not encode multiple isoforms. Many genes annotated in *D*. *melanogaster* as maternally deposited (vs. zygotically expressed or both maternal and zygotic) [32] such as *bbc*, a phosphotransferase, and *Dhc64C*, a gene with ATPase activity, have multiple maternally deposited isoforms, as alternative splicing is detected before zygotic expression begins in embryos of *D*. *pseudoobscura* and *D*. *miranda*.

Of maternal, zygotic, and both maternal and zygotic genes defined in *D*. *melanogaster* [32] for which we recovered orthologs in all four species, zygotic genes show the lowest proportion of AS. However, genes annotated as being in one category in *D*. *melanogaster* are not necessarily in the same category in other species (e.g. a maternal gene in *D*. *melanogaster* may be maternal + zygotic in *D*. *pseudoobscura*). We therefore simply distinguished between genes that are maternally deposited (and that may or may not also show zygotic expression) as genes expressed at developmental stage 2 and zygotic genes (genes not expressed at stage 2 but expressed at later stages of embryonic development) for which we recovered orthologs in *D*. *pseudoobscura* and *D*. *miranda*. Maternally deposited genes show a higher proportion of AS than zygotic genes in both species (*D*. *miranda*: 180 of 2173 (8.3%) maternally deposited genes alternatively spliced vs. 7 of 647 (1.1%) zygotic genes alternatively spliced; *D*. *pseudoobscura*: 159 of 2149 (7.4%) maternally deposited genes alternatively spliced vs. 2 of 704 (0.3%) zygotic genes alternatively spliced). Thus, maternally deposited mRNA may comprise higher transcriptome diversity than previously appreciated, consistent with the abundance of alternatively spliced transcripts that we detect in ovaries. Among the zygotic genes, we confirmed sex-specific alternative splicing events of the *Sxl* gene, the master sex determining gene of *Drosophila*, during early embryogenesis in *D*. *pseudoobscura* and *D*. *miranda* (see below).

**Fig 3:**
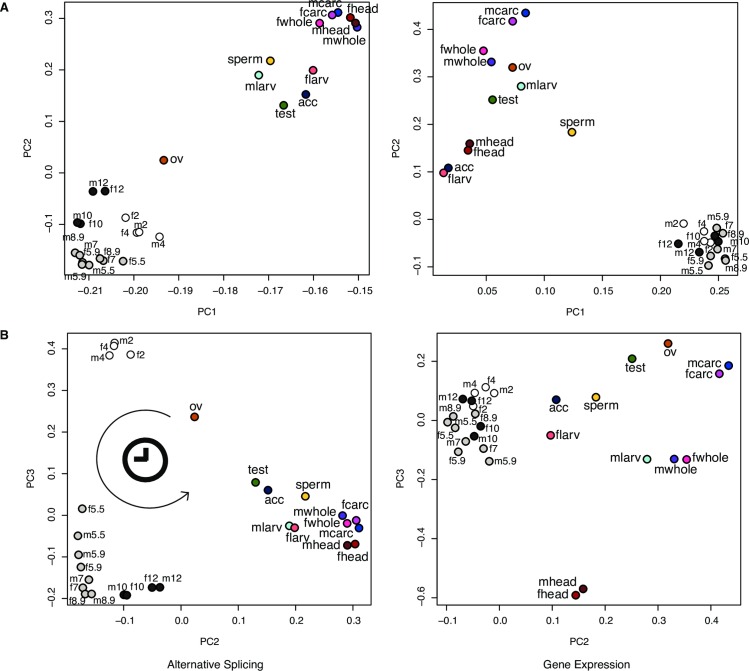
PCAs based on AS and GE. Alternative splicing (left column) and gene expression (right column) profiles for *D*. *pseudoobscura*. The R function *prcomp* was used to perform the PCAs. (A) PC1 (AS: 57.9% of the variance & GE: 50.1% of the variance) and PC2 (AS: 10.7% of the variance & GE: 12.4% of the variance). (B) PC2 and PC3 (AS: 4.5% of the variance & GE: 7.5% of the variance). “f” = female; “m” = male; “5.5” = mid stage 5; “5.9” = late stage 5; “8.9” = late stage 8; “carc” = carcass; “ov” = ovary; “sperm” = spermatheca; “larv” = 3rd instar larva; “test” = testis; “acc” = accessory gland

PCA analysis of splicing over embryonic development shows that different embryonic stages have distinct splicing profiles, which form a clock-like pattern in the PCA plot corresponding to the developmental time course (**Fig 3**; shown is *D*. *pseudoobscura*, and similar trends are seen for the other species; see **S1**–**S3 Figs**). This differs from gene expression, which does not differentiate the different embryonic stages to the same degree as splicing (**Fig 3**). Remarkably, in PCAs based on AS profiles, ovary is the closest of all post-embryonic tissues to embryonic stages while PCAs based on GE do not show ovary as being particularly close to embryonic stages. This together with the observation that both ovary and prezygotic embryos express few genes but have a great proportion of alternatively spliced transcripts (**Fig 2**) suggests that AS of maternally deposited transcripts plays an important role in early embryonic development.

### Temporal and spatial expression dynamics across species

We compared gene expression levels and alternative splicing profiles across developmental stages (between *D*. *pseudoobscura* and *D*. *miranda*) and tissues (among *D*. *pseudoobscura*, *D*. *miranda*, *D*. *albomicans*, and *D*. *nasuta*). We recovered 3,005 orthologous genes among all four species (and 6,707 between *D*. *pseudoobscura* and *D*. *miranda*) and 472 orthologous exons expressed and annotated as alternatively spliced in at least one sample in all four species (and 1,122 between *D*. *pseudoobscura* and *D*. *miranda*). Recovery of fewer genes and exons among all four species compared to just *D*. *pseudoobscura* and *D*. *miranda* is expected, due to greater divergence times and less power to identify genomic features in species with lower quality genome assemblies and fewer RNA-seq data (i.e. *D*. *albomicans* and *D*. *nasuta*, see **S1 Table**). As a measure of gene expression for a gene in the interspecific comparisons, we used TPM values (Transcripts Per Million), while alternative splicing was quantified using Ψ (Percent of exons Spliced In/“PSI”), the proportion of isoforms containing an alternatively spliced exon (or retained intron).

**Fig 4:**
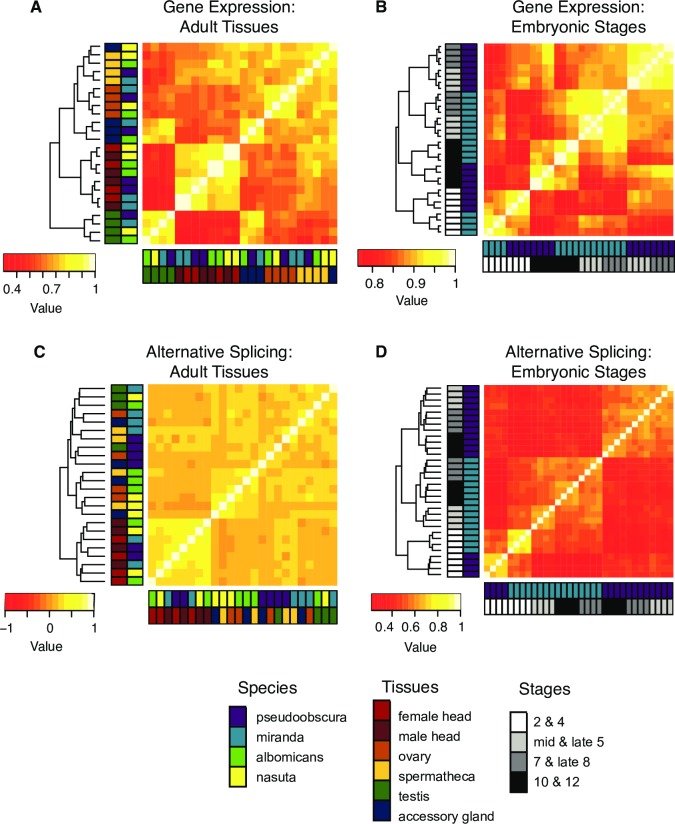
Correlations of gene expression versus alternative splicing. Spearman correlations based on gene expression (TPM) for orthologous genes in (A) adult tissues (n = 3,005) and (B) embryonic stages (n = 6,707) (mean of three replicates per sex/stage). Spearman correlations based on alternative splicing (Ψ) for orthologous exons (C) expressed in all species and annotated as alternatively spliced in at least one sample in adult tissues (n = 472) and (D) expressed in both species and annotated as alternatively spliced in at least one sample in embryonic stages (n = 1,122).

When clustering adult samples on the basis of how gene expression levels correlate in pairwise comparisons, there is no strong species-specific clustering, and clustering is almost completely tissue-specific (**Fig 4A**). Clustering embryonic stages based on gene expression levels (**Fig 4B**) results in stage-specific clustering across embryogenesis, and within broad groups of stages, species-specific clustering. Gene expression during early embryogenesis is conserved over particular groups of developmental stages, and in adults, tissue-specific expression patterns dominate.

When clustering adult alternative splicing profiles using Ψ, species-based clustering tends to be stronger than tissue-based clustering (**Fig 4C**). During embryonic development, samples segregate broadly by developmental stage: prezygotic (2 and 4) and postzygotic (mid stage 5– stage 12) stages cluster, and within those categories, the samples cluster by species (**Fig 4D**). The more tissue-based clustering in adults observed for gene expression and the stronger species-based clustering in all samples based on splicing (for gene expression, stages 2 & 4 and stages 10 & 12 cluster by developmental stage, while only the prezygotic stages 2 and 4 cluster for splicing) is consistent with the strong species-specific clustering observed for splicing and “tissue-dominated clustering” observed for gene expression among vertebrates [7]. A comparison between gene expression and splicing has not been done over embryonic development in vertebrates, and it will be of great interest to see if embryogenesis in vertebrates follows the same patterns we see in *Drosophila*.

### Sex-biased and sex-specific alternative splicing

**Fig 5:**
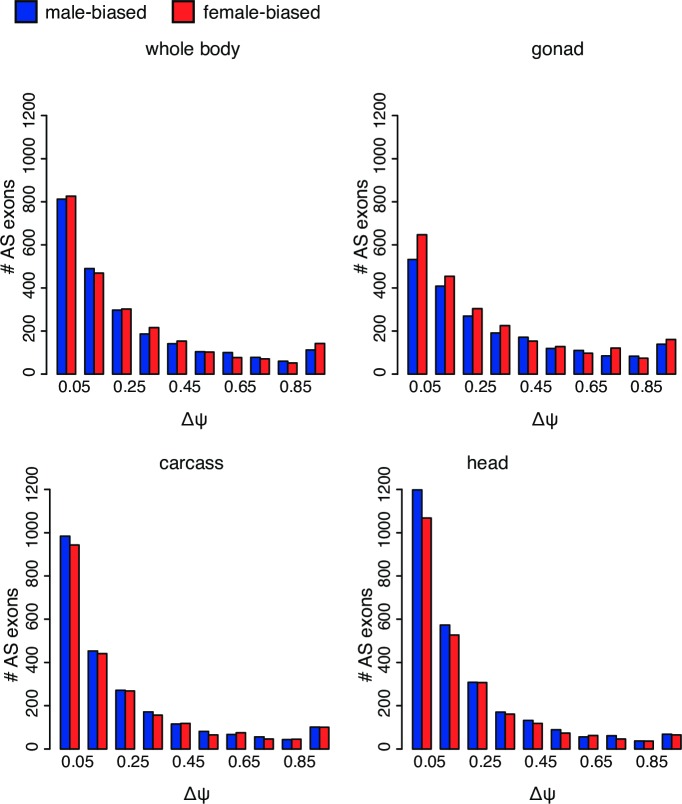
Sex-biased splicing in *D*. *pseudoobscura*. Comparisons of **Δ**Ψ distributions are between males and females for whole body (top left), gonad (ovary and testis, top right), carcass (bottom left), and head (bottom right). The x-axis gives ΔΨ values and the y-axis shows the number of sex-biased exons. Red bars represent female-biased exons (Ψ_female_− Ψ_male_ > 0) and blue bars represent male-based exons (Ψ_male_− Ψ_female_ > 0).

Alternative splicing mediates sex determination in Drosophila, and our RNA-Seq data confirm sex-biased splicing of genes involved in sex determination. For example, we detect one of the exons included in males and spliced out in females for *Sxl* in the two *Drosophila* species for which we have developmental expression data (**S4 Fig**). We used ΔΨ values (ΔΨ = |Ψ_female_ - Ψ_male_|) to assess sex-biased alternative splicing in various male and female tissues and stages (**Fig 5**), and find sex-biased exons from genes previously observed to have sex-biased isoforms [8], such as the male-biased isoform of *thin*, a gene involved in protein ubiquitination. Proportionally, gonads show more pronounced sex-biased splicing, and the splicing pattern for gonadectomized whole flies is skewed towards weaker sex-biased splicing, for all species (**Fig 5**, **S5**–**S7 Figs)**. For example, in *D*. *pseudoobscura* carcasses, 8.5% of alternatively spliced exons are strongly sex-biased (ΔΨ > 0.7) while in *D*. *pseudoobscura* gonads (ovary and testis), 14.8% of alternatively spliced exons are strongly sex-biased (**Fig 5**). Similar patterns are also seen for the other three species, with alternatively spliced exons being more strongly sex-biased in gonads (11.5–18.3%) than in carcasses (7.6–16.4%; see **S5**–**S7 Figs** for ΔΨ analyses for other species). As mentioned, heads show the highest number of AS events, but most of them show only very weak sex-bias. For example, in *D*. *pseudoobscura* heads, only 56.0% of sex-biased exons have a ΔΨ > 0.10, while in gonads 73.6% of sex-biased exons have a ΔΨ > 0.10 (**Fig 5**). Conversely, more *D*. *pseudoobscura* exons are strongly sex-biased (ΔΨ > 0.7) in gonads (14.8%) than in heads (6.1%). Similar patterns are apparent in the other three species: within *D*. *miranda* and *D*. *nasuta*, more exons have a ΔΨ > 0.10 in gonads (77.5% and 74.1%, respectively) than in heads (61.9% and 64.3%), while those fractions are similar for *D*. *albomicans* (70.8% in gonads vs. 71.4% in heads). However, for all three species, gonads have a higher proportion of strongly sex-biased (ΔΨ > 0.7) exons (11.5–18.3%) than heads (4.78%-6.9%) (**S5**–**S7 Figs**). Thus, most of the sex-biased AS found in flies can be attributed to AS in gonads.

## Discussion

Consistent with previous studies, we find that AS significantly contributes to increasing the transcriptome diversity in all *Drosophila* species examined, and approximately 40% of genes are alternatively spliced. In every species studied, we find that head tissue harbors the largest number of alternatively spliced exons, and the highest fraction of expressed genes that are alternatively spliced is found in ovary. High transcriptional diversity has also been reported in brain tissues of mammals and vertebrates [6, 7, 30, 31], and it will be interesting to see how ovarian transcriptional diversity compares to head in other species as well.

While males generally show a higher number of genes expressed than females, female-specific tissues (ovary and spermatheca) have the highest percentage of alternatively spliced genes. Thus, male tissues may increase their transcriptome diversity by expressing more genes, while female tissues increase the number of transcripts through AS.

Gene expression levels have been found to cluster by tissue across different mammalian and vertebrate species[6, 7]. Our analysis of *Drosophila* adult tissues reveals similar strong tissue-specific clustering, with only one exception (*D*. *nasuta* accessory gland clustering with *D*. *nasuta* spermatheca). This is interesting because the mammalian and vertebrate studies mostly examined somatic tissues (such as liver or kidney) with the exception of testis. In contrast, most of our samples (with the exception of male and female head) contain gonad tissue (ovary and testis) or sex-specific tissues (spermatheca and accessory gland), which have been shown to be rapidly evolving [33–37]. However, while sex-specific tissues may evolve more rapidly than somatic tissues, our analyses show that gene expression profiles of those tissues are conserved among species. Note that the vertebrate studies similarly compare species with highly varying divergence times (between 6 and 350 MY [6]) as our *Drosophila* study does (between 0.1 [22] and 60 MY [23]). None of the evolutionary patterns reported differ between distantly- and closely-related species–i.e. species- vs. tissue-specific clustering for alternative splicing vs. gene expression does not differ between closely- vs. distantly-related species (see **Fig 4**). Thus, divergence time does not appear to affect any of our analysis.

Similar to the findings in mammals and vertebrates, we see strong species-specific clustering for alternative splicing in *Drosophila*. One caveat is that the one purely somatic tissue, head, clusters by tissue. This could mean that AS in gonad tissue and sex-specific tissues may be evolving more rapidly, and AS may be more conserved in somatic tissue. Also, the lack of lineage-specific clustering in head may be due to shorter divergence times in flies (though the number of generations is probably higher). Our interspecies AS analyses focused on all exons annotated as alternatively spliced in at least one sample (for adult tissue splicing analyses, n = 472 exons). However, we only recovered 49 exons alternatively spliced in at least one sample in all four species and 330 alternatively spliced exons unique to a species. Note that we used stringent cut-offs to identify orthologous alternatively spliced exons, that is, all exons in our interspecific analyses have an FPKM > 1 in at least one sample per species. While it is possible that we may miss some lowly expressed shared alternatively spliced exons, this suggests that many exons in flies that are alternatively spliced in one species are constitutive in all the others. Species-specific splicing differences in *Drosophila* therefore may be based mostly on the binary category of whether an exon is alternatively spliced or constitutive rather than on differences in Ψ values of orthologous exons among species.

While species-specific clustering for alternative splicing is consistent with lineage-specific adaptive evolution [6, 7], it may also support the hypothesis that much splicing is due to erroneous splice site choice, producing non-functional isoforms targeted for degradation/nonsense-mediated decay [38, 39]. These presumably deleterious splicing events would therefore be unlikely to be evolutionarily conserved among species.

We find that when comparing all tissues, tissue differences in gene expression patterns in adults are more dominant than sex differences; for example, male heads are closer to female heads than other male tissues. Note that head is a composite structure, but this finding is consistent with previous studies that found sex differences in *D*. *melanogaster* brain gene expression to be low [40]. When restricting our analysis to sex-specific tissues (ovary, spermatheca, testis, accessory gland), we see clustering primarily by sex and within sex, clustering by tissue (**S8 Fig**). We also find evidence for considerable sex-specific splicing; however, most of the extreme differences in splicing between sexes are due to differences in splicing profiles in gonads.

In the middle of embryonic development (mid stage 5—late stage 8), stages cluster by species based both on gene expression and alternative splicing. After embryogenesis, gene expression becomes more conserved among tissues and species-specific clustering is less dominant. Alternative splicing profiles cluster samples by species rather than tissue (with the exception of head) into adulthood, suggesting that alternative splicing has diverged more than gene expression levels among *Drosophila* species. Note that we only had embryonic data for two species, and only over the early stages of embryogenesis (i.e. roughly the first half of development), and it will be of great interest to see whether other species show similar patterns during early development.

We show that females deposit a diversity of isoforms into the egg, and maternally deposited genes show a higher proportion of AS than zygotic genes in both species investigated. This relates to a well-known property of early development of *D*. *melanogaster*, that the first zygotic transcripts tend to be short and lacking introns [41]. The shortness/low complexity of early transcripts is thought to reflect time constraints on producing longer (or spliced) transcripts in quickly dividing early embryos [42, 43] during development [44]. Thus, longer and more complex zygotically expressed transcripts may not reach high levels of expression during short mitotic cycles due to the time required for full transcription and splicing. In *D*. *melanogaster*, zygotic genome activation starts from intronless genes [45] and RNA-seq coverage along transcripts show patterns consistent with intron delay and an inability to fully transcribe long transcripts [46]. On the other hand, maternally deposited mRNAs may be especially long and complex (i.e. many introns and alternatively spliced exons) because zygotes may not be able to produce them early on [46].

Similar to findings in *D*. *melanogaster* [45, 46], we do see evidence of shorter genes and fewer introns during early zygotic transcription. In particular, comparisons of the two earlier stages that consist of mostly maternally deposited transcripts (stage 2 and stage 4) with later embryonic stages where zygotic transcription is occurring (mid stage 5 –stage 12) show that transcripts present during the earlier stages are longer and have more exons than those expressed during later stages (**S9 Fig**).

## Methods

### Data

The accession numbers for the previously publicly accessible RNA-seq datasets are listed in **S2 Table**.

The remaining samples are RNA-seq datasets generated by us from *D*. *pseudoobscura* (MV25) (male and female 3^rd^ instar larva, spermatheca), *D*. *miranda* (MSH22) (male and female head, spermatheca), *D*. *albomicans* (KM55) (male and female gonadectomized carcass, male and female head, ovary, spermatheca, testis, accessory gland, male and female 3^rd^ instar larva), and *D*. *nasuta* (15112–1781.00) (male and female gonadectomized carcass, male and female head, ovary, spermatheca, testis, accessory gland, male and female 3^rd^ instar larva). We extracted total RNA from whole body or dissected tissues (Qiagen) and prepared RNA-seq libraries following the standard Illumina protocol. Briefly, we used Dynal oligo(dT) beads (Invitrogen) to isolate poly(A) mRNA from the total RNA samples. We then fragmented the mRNA by using the RNA fragmentation kit from Ambion, followed by first- and second-strand cDNA synthesis using random hexamer primers (Invitrogen). We complemented the cDNA synthesis by an end repair reaction using T4 DNA polymerase and Klenow DNA polymerase for 30 min at 20°C. We then added a single A base to the cDNA molecules by using 3'-to-5' exonuclease and ligated the Illumina adapter. The fragments were subjected to size selection on a 2% gel and purification (Qiagen). We finally amplified the cDNA fragments by PCR reaction and examined the libraries by Bioanalyzer (Aglient). Paired-end cDNA sequencing was performed on the Illumina HiSeq 2000 at the Vincent J. Coates Genomics Sequencing Laboratory at the University of California, Berkeley. This work used the Vincent J. Coates Genomics Sequencing Laboratory at UC Berkeley, supported by NIH S10 Instrumentation Grants S10RR029668 and S10RR027303.

More information on how to access this data can be found in DATA ACCESS.

### Within-species analyses

Within-species analyses are analyses where only samples within a species were directly compared (**Figs 1**–**3**, **Fig 5**, **S1**–**S7 Figs**, **S9A and S9B Fig**, **S9E and S9F Fig** and **S4 Table**). We used seqtk (https://aur.archlinux.org/packages/seqtk-git) to randomly choose paired-end RNA-seq reads so that within each species, each sample used had the same number of reads (**S1 Table**). The pooled data from all four species had a total of 957,749,296 reads. For samples with read lengths > 50 bp, we used read quality data from FastQC (http://www.bioinformatics.bbsrc.ac.uk/projects/fastqc) to determine cutoffs and trimmed the reads to 50 bp.

We used bowtie2-build and TopHat v2.0.13 [47] to map the reads to each genome (*D*. *pseudoobscura* Release 3.1 downloaded from http://www.flybase.org, *D*. *miranda* assembly [27], *D*. *albomicans* assembly [26], *D*. *nasuta* assembly, unpublished, **S3 Table**) using the parameters —b2-sensitive,—coverage-search, and—microexon-search and then ran Cufflinks v2.1.1 [48] without a reference annotation on each sample. For each species, we used cuffmerge to combine all of the cufflinks-generated annotations to create a master annotation. We used cuffdiff [49] with this master annotation to obtain expression data for each sample. We use the “commonly adopted arbitrary inclusion threshold” of FPKM = 1 [50] that is used in other *Drosophila* gene expression studies [18].

We used picard v1.106 (http://picard.sourceforge.net) to obtain insert size mean and standard deviation for each sample. We used MATS [51, 52] to on one hand detect and annotate putative AS events from our aligned RNA-seq data (bam files) and cuffmerge annotations. MATS was also used to get Ψ (“PSI”, Percent Spliced In) values for each alternatively spliced exon by running pairwise comparisons between all samples within each species and taking an average Ψ value for each exon calculated using reads on target and junction counts, excluding samples for which the exon was not classified as alternatively spliced. The program was run using default parameters except setting the “-analysis” parameter to “P” for our paired-end sequencing reads. The anchor/overhang length was the tophat2 default of 8 bp, so at least eight nucleotides had to map to each end of a given junction. The annotation of AS events by MATS included intron retention events that overlapped with splice sites of other annotated AS events. To validate our pipeline, we also tested a few tissues using an alternative pipeline. We used AltEventFinder [53] to annotate skipped exon alternative splicing events and MISO [4, 54] to compute Ψ values. **S4 Table** shows correlations of the results of both pipelines for post-embryonic tissues in *D*. *miranda* computed in R using the *corr* function.

*heatmap*.*2* was used in R to generate heatmaps, which uses *hclust* to cluster samples. The R function *prcomp* was used to perform the PCAs. The R function *wilcox*.*test* was used to perform Wilcoxon rank sum tests (**S9 Fig**).

Note that for analyses within species, we directly compare datasets subsampled down to the same number of reads (**S1 Table**) that are mapped to the same genome assembly. Therefore, differences in genome assembly quality among the four genomes and the differences in the numbers of reads used among species are not expected to impact our within-species inferences.

### Interspecific comparisons

Interspecific comparisons are analyses in which samples among different species were compared directly (**Fig 4**, **S8 Fig**, **S9C and S9D Fig** and **S10 and S11 Figs**). Pairwise whole genome alignments for each pair of species were done using the software Mercator (https://www.biostat.wisc.edu/~cdewey/mercator/) [55] and MAVID [56]. First we used Mercator to build an orthology map for each pair of species. Then MAVID was used to perform global whole genome alignments. Finally, for each exon/gene in each species, the coordinates of the corresponding ortholog in the other species were determined using the sliceAlignment program (Mercator distribution).

Using pairwise alignments of *D*. *pseudoobscura*, *D*. *miranda*, *D*. *albomicans*, and *D*. *nasuta* to *D*. *melanogaster*, we kept genes/exons aligned with >0.5 overlap between all pairs. We kept all genes/exons with 1:1 orthology. If there were multiple genes/exons from one species that aligned to the same *D*. *melanogaster* gene/exon, we kept the pair with the highest overlap score. If the highest overlap score was shared between the *D*. *melanogaster* gene/exon and more than one gene/exon from the other species, we did not use the gene(s)/exon(s)in our analysis. If there were multiple *D*. *melanogaster* genes/exons that aligned to the same gene/exon from the other species, we kept the pair with the highest overlap score. If the highest overlap score was shared between one gene/exon from the other species and more than one *D*. *melanogaster* gene/exon, we did not use the gene(s)/exon(s) in our analysis. For comparisons over embryonic development, this left us 6,707 genes with 1:1 orthologous relationships between *D*. *pseudoobscura* and *D*. *miranda* and 1,122 exons with 1:1 orthologous relationships between *D*. *pseudoobscura* and *D*. *miranda* that were also annotated as alternatively spliced in at least one sample and expressed with an FPKM > 1 in at least one sample per species. For comparisons of post-embryonic tissues, we recovered 3,005 genes with 1:1 orthologous relationships among *D*. *pseudoobscura*, *D*. *miranda*, *D*. *albomicans*, and *D*. *nasuta* and 472 exons with 1:1 orthologous relationships among the four species that were also annotated as alternatively spliced in at least one sample and expressed with an FPKM > 1 in at least one sample per species.

We used orthology information to create annotations for each species containing only genes orthologous among all species (*D*. *pseudoobscura*, *D*. *miranda*, *D*. *albomicans*, and *D*. *nasuta* for comparisons of post-embryonic samples; *D*. *pseudoobscura* and *D*. *miranda* for comparisons of embryonic samples) and ran kallisto [57] using these annotations. We used TPM (Transcripts Per Million) values from each sample for each gene for our interspecies gene expression analysis. We used sleuth [57] to normalize TPM values between samples and to compute Jensen-Shannon divergence between samples.

We used bowtie2-build and TopHat v2.0.13 [47] to map the reads to each genome (*D*. *pseudoobscura* Release 3.1 downloaded from http://www.flybase.org, *D*. *miranda* assembly [27], *D*. *albomicans* assembly [26], *D*. *nasuta* assembly (unpublished, **S3 Table**) using the parameters —b2-sensitive,—coverage-search, and—microexon-search and then ran Cufflinks v2.1.1 [48] using the–G parameter and the reference annotations used for orthology analysis on each sample.

For interspecies splicing analyses, we ran MATS [51, 52] to on one hand detect and annotate putative AS events from our aligned RNA-seq data (bam files) and orthology annotations. MATS was also used to get Ψ (“PSI”, Percent Spliced In) values for each alternatively spliced exon. The program was run using the same parameters as for the “within-species” analysis. MATS compares splicing in samples pairwise, and we compared all samples within species that shared the same read lengths. Spermatheca reads from *D*. *nasuta* and *D*. *albomicans* were trimmed to 76 base pairs (the size of all other reads in these species). In each sample, we took an average Ψ value for each exon calculated using reads on target and junction counts, excluding samples for which the exon was not classified as alternatively spliced. For exons alternatively spliced in some samples but not others, we looked at the expression calculated for that exon in cufflinks. If the exon had an FPKM value < 1 and the upstream or downstream exons had an FPKM value > 1, the exon was assigned a Ψ value of 0. If the exon had an FPKM value > 1, the exon was assigned a Ψ value of 1. If the exon had an FPKM value < 1 and the upstream and downstream exons had an FPKM value < 1, the exon was not assigned a Ψ value.

*heatmap*.*2* was used in R to generate heatmaps, which uses *hclust* to cluster samples. We computed Spearman (**Fig 4, S8 Fig**) and Pearson (**S10 Fig**) correlations for GE and AS in adult tissues and embryonic stages in R using the *corr* function. Jensen-Shannon divergence of GE (**S11 Fig**) was computed using sleuth[57].

For analyses among species, we directly compare only orthologous genes and exons recovered in all four species. Therefore, our inferences are limited by the least complete genome assembly and annotation. We did not subsample datasets for our interspecies analyses (**S1 Table**), but instead required that each orthologous gene or exon be expressed at FPKM > 1 in at least one sample per species to be included in our analyses. We consider our interspecies analysis conservative; we may be underestimating the number of orthologous genes and comparable splicing events. However, due to our approach, we do not expect a considerable number of splicing events or expressed genes to be erroneously identified as species-specific.

## Supporting Information

S1 Fig PCAs based on AS and GE for *D*. *miranda*. Alternative splicing (left column) and gene expression (right column) profiles for *D*. *miranda*. The R function *prcomp* was used to perform the PCAs. (top) PC1 (AS: 74.7% of the variance & GE: 98.4% of the variance) and PC2 (AS: 7.4% of the variance & GE: 1.5% of the variance). (bottom) PC2 (AS: 7.4% of the variance & GE: 1.5% of the variance) and PC3 (AS: 4.3% of the variance & GE: 0.05% of the variance). “f” = female; “m” = male; “5.5” = mid stage 5; “5.9” = late stage 5; “8.9” = late stage 8; “carc” = carcass; “ov” = ovary; “sperm” = spermatheca; “larv” = 3rd instar larva; “test” = testis; “acc” = accessory gland
(TIF)

S2 Fig PCAs based on AS and GE for *D*. *albomicans*. Alternative splicing (left column) and gene expression (right column) profiles for *D*. *albomicans*. The R function *prcomp* was used to perform the PCAs. (top) PC1 (AS: 54.7% of the variance & GE: 40.5% of the variance) and PC2 (AS: 7.6% of the variance & GE: 23.7% of the variance). (bottom) PC2 (AS: 7.6% of the variance & GE: 23.7% of the variance) and PC3 (AS: 6.0% of the variance & GE: 9.2% of the variance). “carc” = carcass; “ov” = ovary; “sperm” = spermatheca; “larv” = 3rd instar larva; “test” = testis; “acc” = accessory gland
(TIF)

S3 Fig PCAs based on AS and GE for *D*. *nasuta*. Alternative splicing (left column) and gene expression (right column) profiles for *D*. *nasuta*. The R function *prcomp* was used to perform the PCAs. (top) PC1 (AS: 54.3% of the variance & GE: 97.3% of the variance) and PC2 (AS: 7.7% of the variance & GE: 2.5% of the variance). (bottom) PC2 (AS: 7.7% of the variance & GE: 2.5% of the variance) and PC3 (AS: 5.3% of the variance & GE: 0.2% of the variance). “carc” = carcass; “ov” = ovary; “sperm” = spermatheca; “larv” = 3rd instar larva; “test” = testis; “acc” = accessory gland
(TIF)

S4 Fig *Sxl* expression in embryonic stages. Exon 2 is spliced in in males (blue) and skipped in females (red). We used IGV[58, 59] to visualize *Sxl* expression. “5.5” = mid stage 5; “5.9” = late stage 5; “8.9” = late stage 8.
(TIF)

S5 Fig Sex-biased splicing in *D*. *miranda* as described by ΔΨ distributions. Comparisons are between males and females for whole body (top left), gonad (ovary and testis, top right), carcass (bottom left), and head (bottom right). The x-axis represents **Δ**Ψ values and the y-axis represents the number of sex-biased exons. Red bars represent female-biased exons (Ψ_female_− Ψ_male_ >0) and blue bars represent male-based exons (Ψ_male_− Ψ_female_ >0).
(TIF)

S6 Fig Sex-biased splicing in *D*. *albomicans* as described by ΔΨ distributions. Comparisons are between males and females for whole body (top left), gonad (ovary and testis, top right), carcass (bottom left), and head (bottom right). The x-axis represents **Δ**Ψ values and the y-axis represents the number of sex-biased exons. Red bars represent female-biased exons (Ψ_female_− Ψ_male_ >0) and blue bars represent male-based exons (Ψ_male_− Ψ_female_ >0).
(TIF)

S7 Fig Sex-biased splicing in *D*. *nasuta* as described by ΔΨ distributions. Comparisons are between males and females for whole body (top left), gonad (ovary and testis, top right), carcass (bottom left), and head (bottom right). The x-axis represents **Δ**Ψ values and the y-axis represents the number of sex-biased exons. Red bars represent female-biased exons (Ψ_female_− Ψ_male_ >0) and blue bars represent male-based exons (Ψ_male_− Ψ_female_ >0).
(TIF)

S8 Fig Spearman correlations based on GE in adult tissues not including head. Spearman correlations based on gene expression (TPM) for genes orthologous in adult tissues (n = 3005) not including male and female head.
(TIF)

S9 Fig Boxplots comparing length and exon number of expressed genes between early (stage 2-stage 4) and later (mid stage 5 –stage 12) embryonic stages. Log_2_(length) of genes expressed in early (stage 2 –stage 4) and later (mid stage 5 –stage 12) embryonic stages from within species analyses in A) *D*. *pseudoobscura* and B) *D*. *miranda*. Log_2_(length) of orthologous genes from interspecies analysis expressed in early (stage 2 –stage 4) and later (mid stage 5 –stage 12) embryonic stages in C) *D*. *pseudoobscura* and D) *D*. *miranda*. Number of exons per genes expressed in early (stage 2 –stage 4) and later (mid stage 5 –stage 12) embryonic stages from within species analyses in E) *D*. *pseudoobscura* and F) *D*. *miranda*. *P*-values indicate the results of Wilcoxon rank sum tests.
(TIF)

S10 Fig Pearson correlations based on GE and AS. Pearson correlations based on gene expression (TPM) for genes orthologous in adult tissues (n = 3005) (A) and embryonic stages (n = 6707) (B). Pearson correlations based on alternative splicing (Ψ) for exons orthologous and annotated as alternatively spliced in at least one sample in adult tissues (n = 472) (C) and embryonic stages (n = 1122) (D).
(TIF)

S11 Fig Heatmaps based on Jensen-Shannon divergence of GE. Heatmaps based on Jensen-Shannon divergence of gene expression for genes orthologous in adult tissues (n = 3005) (A) and embryonic stages (n = 6707) (B).
(TIF)

S1 Table Data used. The numbers of pairs of paired-end RNA-seq reads used for interspecies analyses, broken down by species and tissue/sex/stage, and intraspecies analysis. “5.5” = mid stage 5; “5.9” = late stage 5; “8.9” = late stage 8
(TIF)

S2 Table SRA identifiers for all RNA-seq datasets publicly available before publication used in this study. (TIF)

S3 Table Information for the unpublished *D*. *nasuta* genome assembly. (TIF)

S4 Table Correlation of skipped exon Ψ values for alternatively spliced exons in post-embryonic *D*. *miranda* tissues computed by two pipelines. The first pipeline, described in Materials and Methods, used MATS to annotate alternatively spliced exons and compute their Ψ values. The second pipeline used AltEventFinder to annotate alternatively spliced exons and MISO to compute their Ψ values.
(TIF)
