# Supplementary figures and images for: Alternative Splicing within and between Drosophila Species, Sexes, Tissues, and Developmental Stages
Source: PLoS Genet. 2016 Dec 9;12(12):e1006464. doi: 10.1371/journal.pgen.1006464 (PMC5147784; doi:10.1371/journal.pgen.1006464)

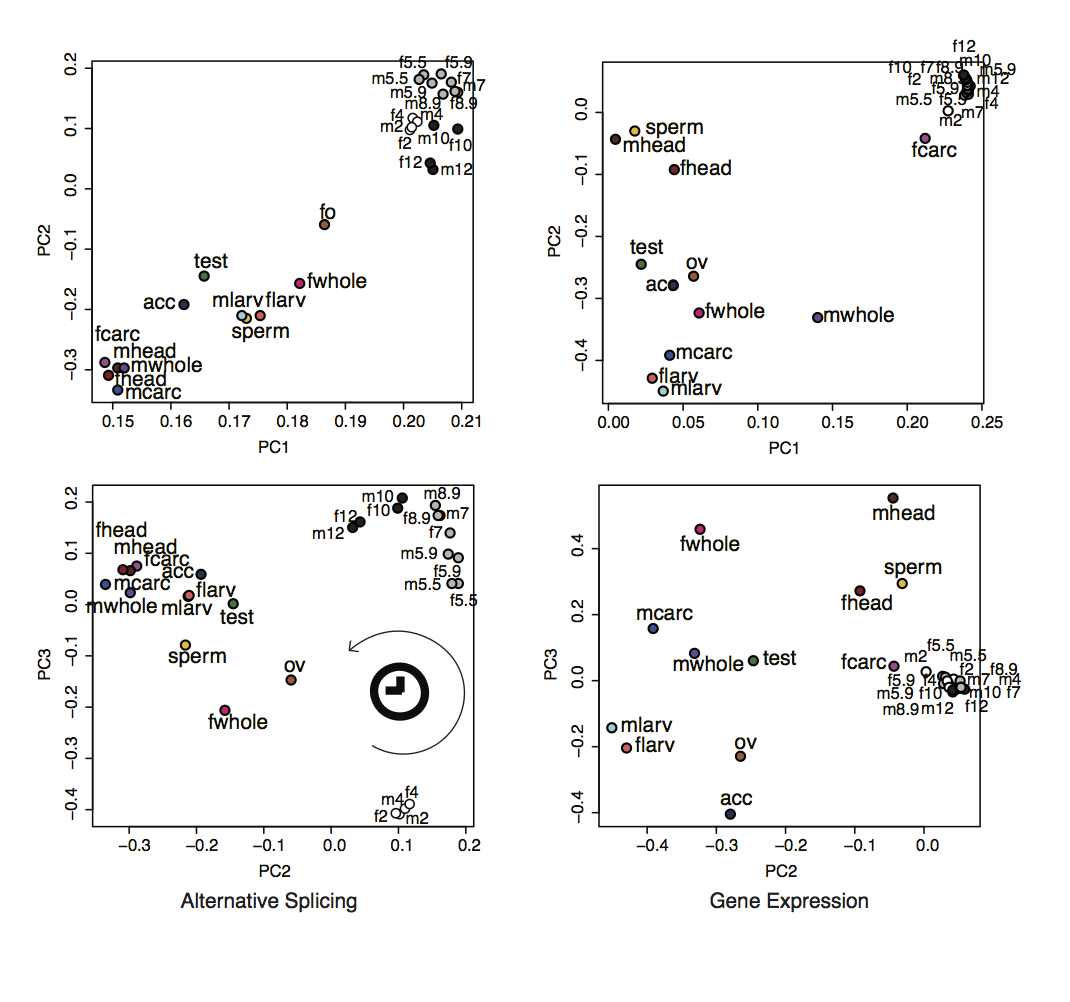

Supplement: S1 Fig — Alternative splicing (left column) and gene expression (right column) profiles for D. miranda. The R function prcomp was used to perform the PCAs. (top) PC1 (AS: 74.7% of the variance & GE: 98.4% of the variance) and PC2 (AS: 7.4% of the variance & GE: 1.5% of the variance). (bottom) PC2 (AS: 7.4% of the variance & GE: 1.5% of the variance) and PC3 (AS: 4.3% of the variance & GE: 0.05% of the variance). “f” = female; “m” = male; “5.5” = mid stage 5; “5.9” = late stage 5; “8.9” = late stage 8; “carc” = carcass; “ov” = ovary; “sperm” = spermatheca; “larv” = 3rd instar larva; “test” = testis; “acc” = accessory gland (TIF) [file pgen.1006464.s001.tif]

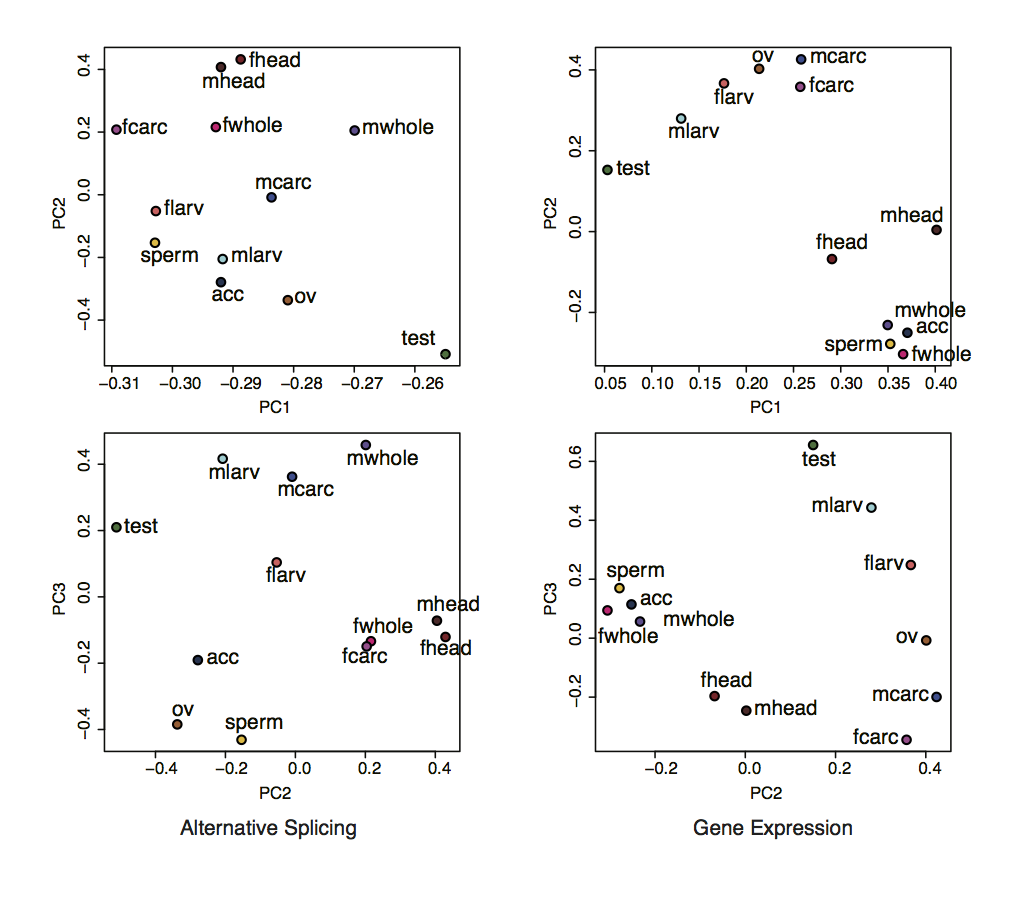

Supplement: S2 Fig — Alternative splicing (left column) and gene expression (right column) profiles for D. albomicans. The R function prcomp was used to perform the PCAs. (top) PC1 (AS: 54.7% of the variance & GE: 40.5% of the variance) and PC2 (AS: 7.6% of the variance & GE: 23.7% of the variance). (bottom) PC2 (AS: 7.6% of the variance & GE: 23.7% of the variance) and PC3 (AS: 6.0% of the variance & GE: 9.2% of the variance). “carc” = carcass; “ov” = ovary; “sperm” = spermatheca; “larv” = 3rd instar larva; “test” = testis; “acc” = accessory gland (TIF) [file pgen.1006464.s002.tif]

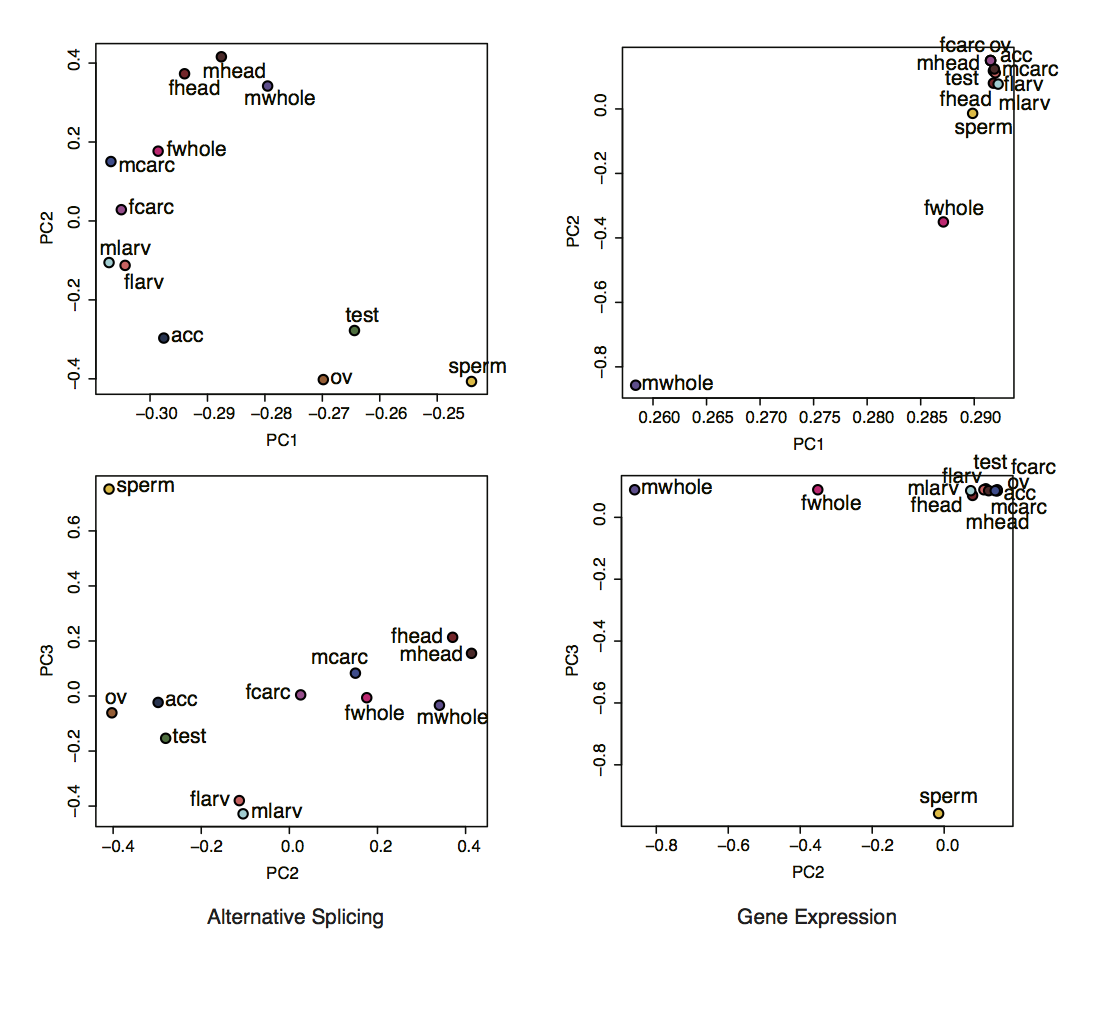

Supplement: S3 Fig — Alternative splicing (left column) and gene expression (right column) profiles for D. nasuta. The R function prcomp was used to perform the PCAs. (top) PC1 (AS: 54.3% of the variance & GE: 97.3% of the variance) and PC2 (AS: 7.7% of the variance & GE: 2.5% of the variance). (bottom) PC2 (AS: 7.7% of the variance & GE: 2.5% of the variance) and PC3 (AS: 5.3% of the variance & GE: 0.2% of the variance). “carc” = carcass; “ov” = ovary; “sperm” = spermatheca; “larv” = 3rd instar larva; “test” = testis; “acc” = accessory gland (TIF) [file pgen.1006464.s003.tif]

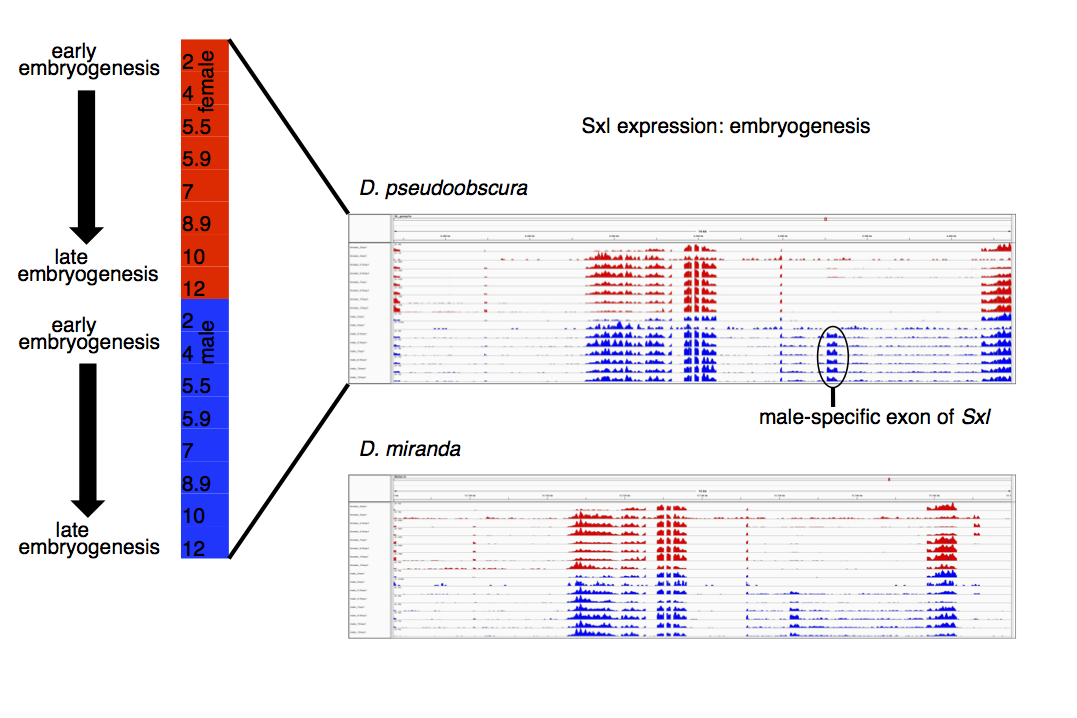

Supplement: S4 Fig — Exon 2 is spliced in in males (blue) and skipped in females (red). We used IGV[58, 59] to visualize Sxl expression. “5.5” = mid stage 5; “5.9” = late stage 5; “8.9” = late stage 8. (TIF) [file pgen.1006464.s004.tif]

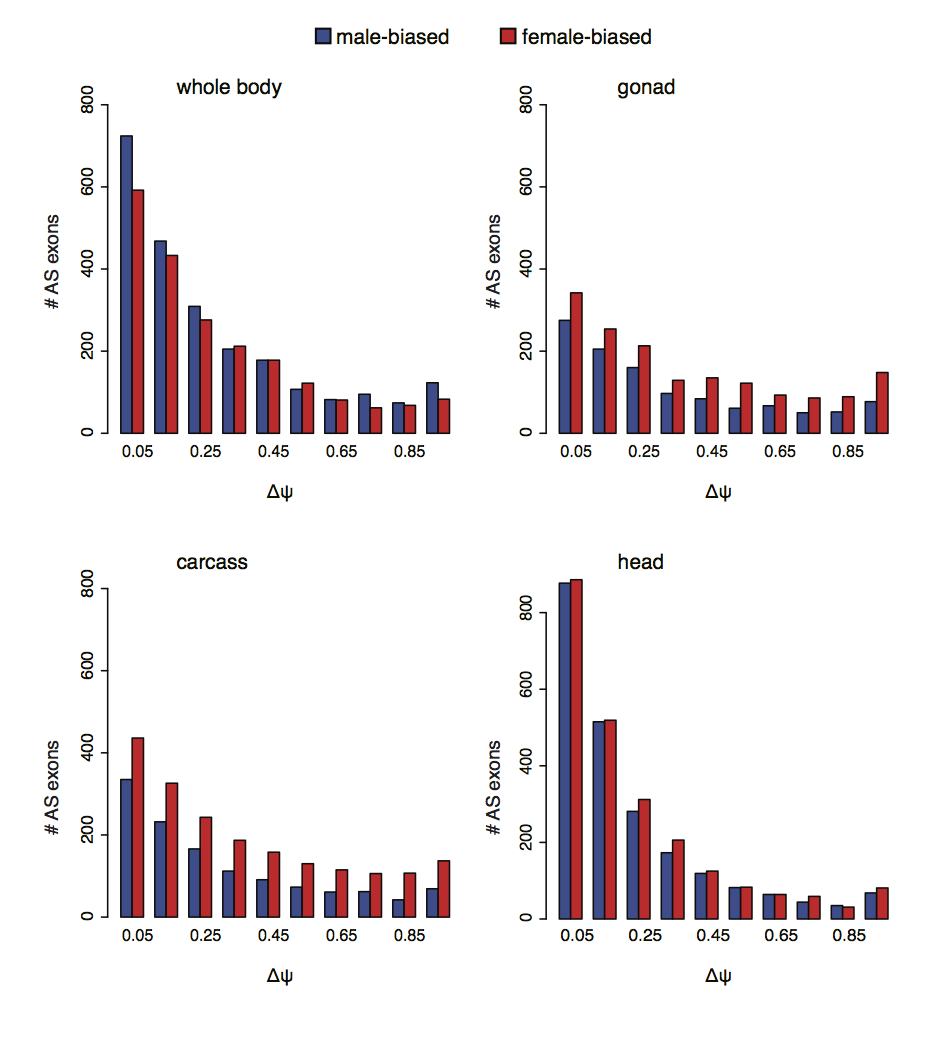

Supplement: S5 Fig — Comparisons are between males and females for whole body (top left), gonad (ovary and testis, top right), carcass (bottom left), and head (bottom right). The x-axis represents ΔΨ values and the y-axis represents the number of sex-biased exons. Red bars represent female-biased exons (Ψfemale− Ψmale >0) and blue bars represent male-based exons (Ψmale− Ψfemale >0). (TIF) [file pgen.1006464.s005.tif]

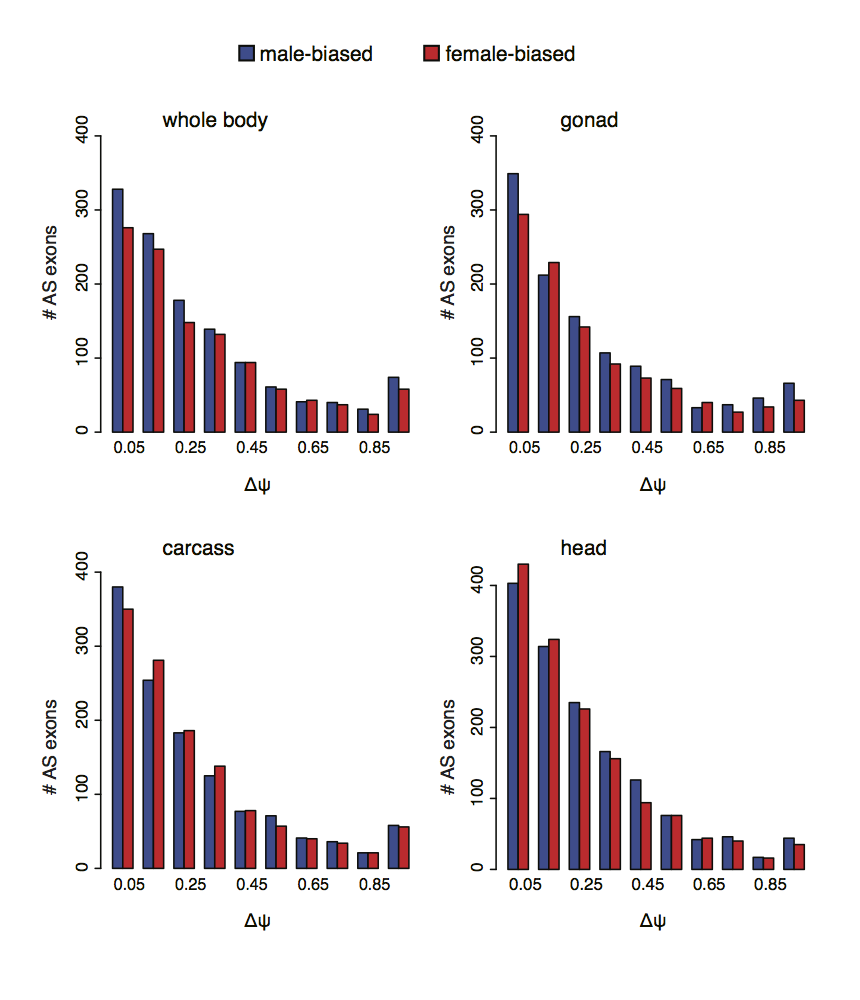

Supplement: S6 Fig — Comparisons are between males and females for whole body (top left), gonad (ovary and testis, top right), carcass (bottom left), and head (bottom right). The x-axis represents ΔΨ values and the y-axis represents the number of sex-biased exons. Red bars represent female-biased exons (Ψfemale− Ψmale >0) and blue bars represent male-based exons (Ψmale− Ψfemale >0). (TIF) [file pgen.1006464.s006.tif]

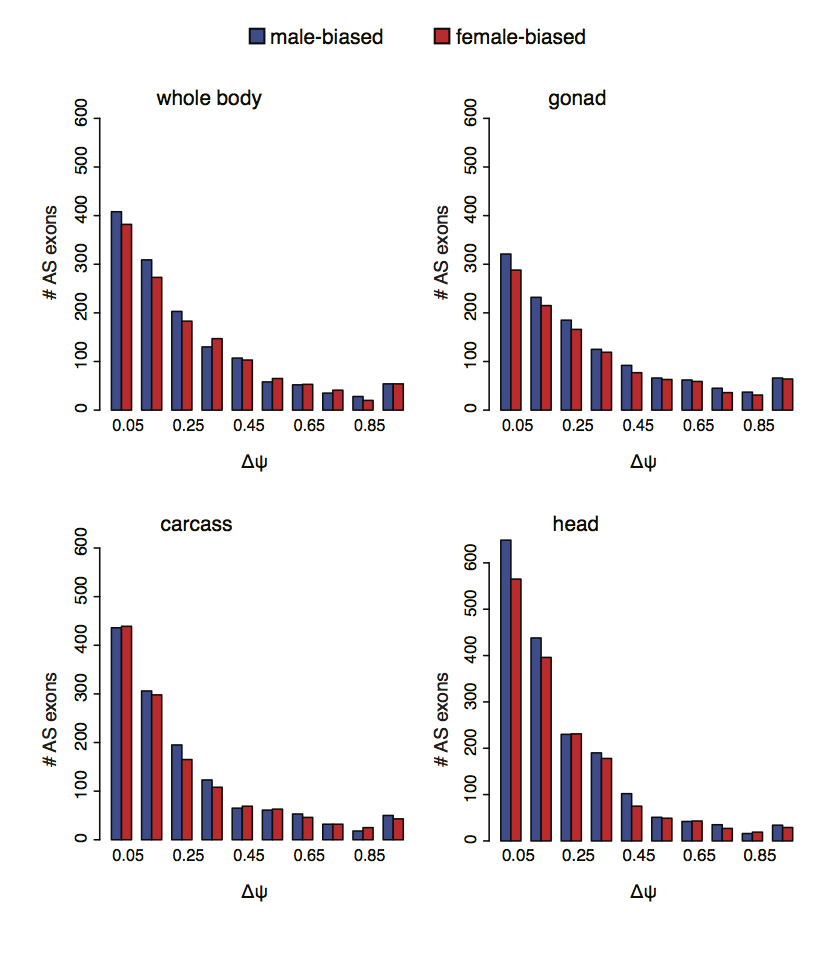

Supplement: S7 Fig — Comparisons are between males and females for whole body (top left), gonad (ovary and testis, top right), carcass (bottom left), and head (bottom right). The x-axis represents ΔΨ values and the y-axis represents the number of sex-biased exons. Red bars represent female-biased exons (Ψfemale− Ψmale >0) and blue bars represent male-based exons (Ψmale− Ψfemale >0). (TIF) [file pgen.1006464.s007.tif]

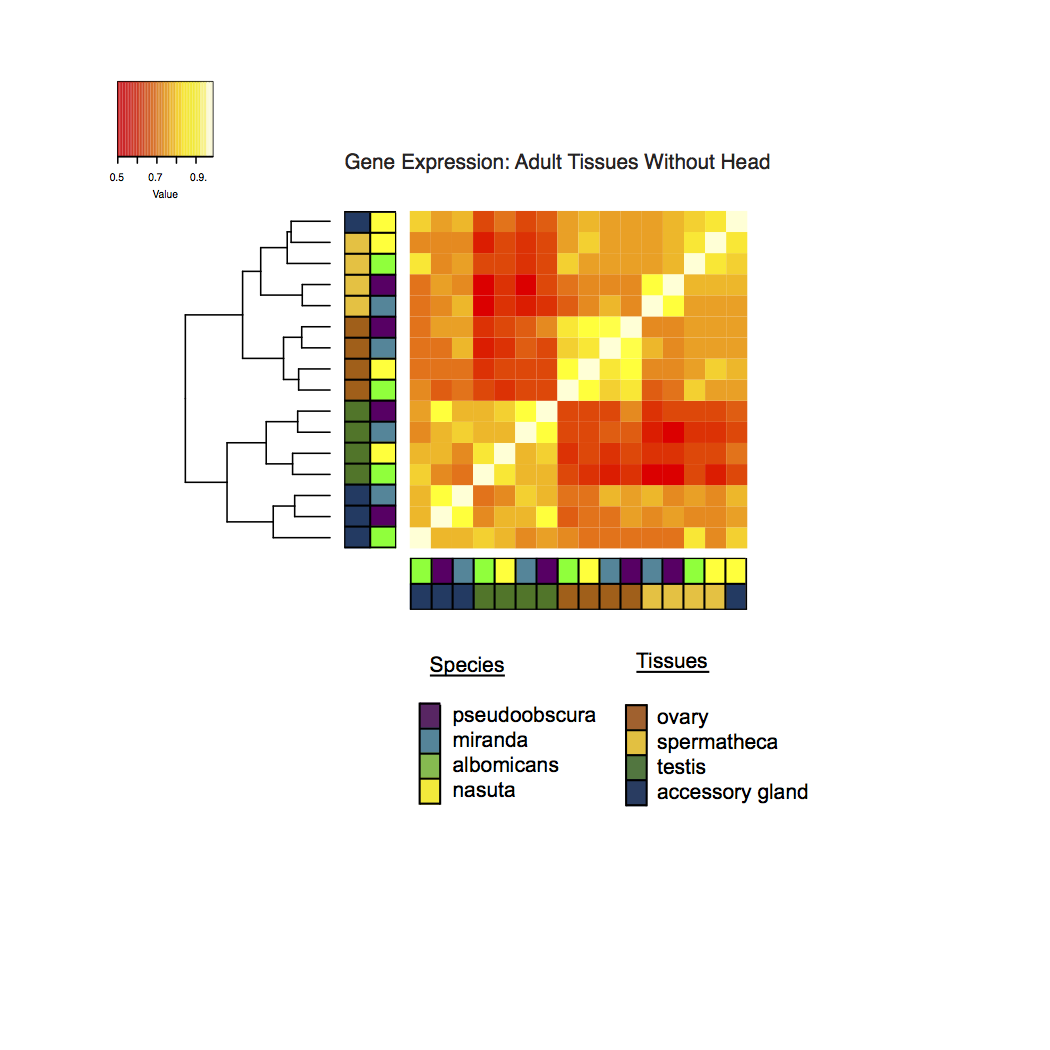

Supplement: S8 Fig — Spearman correlations based on gene expression (TPM) for genes orthologous in adult tissues (n = 3005) not including male and female head. (TIF) [file pgen.1006464.s008.tif]

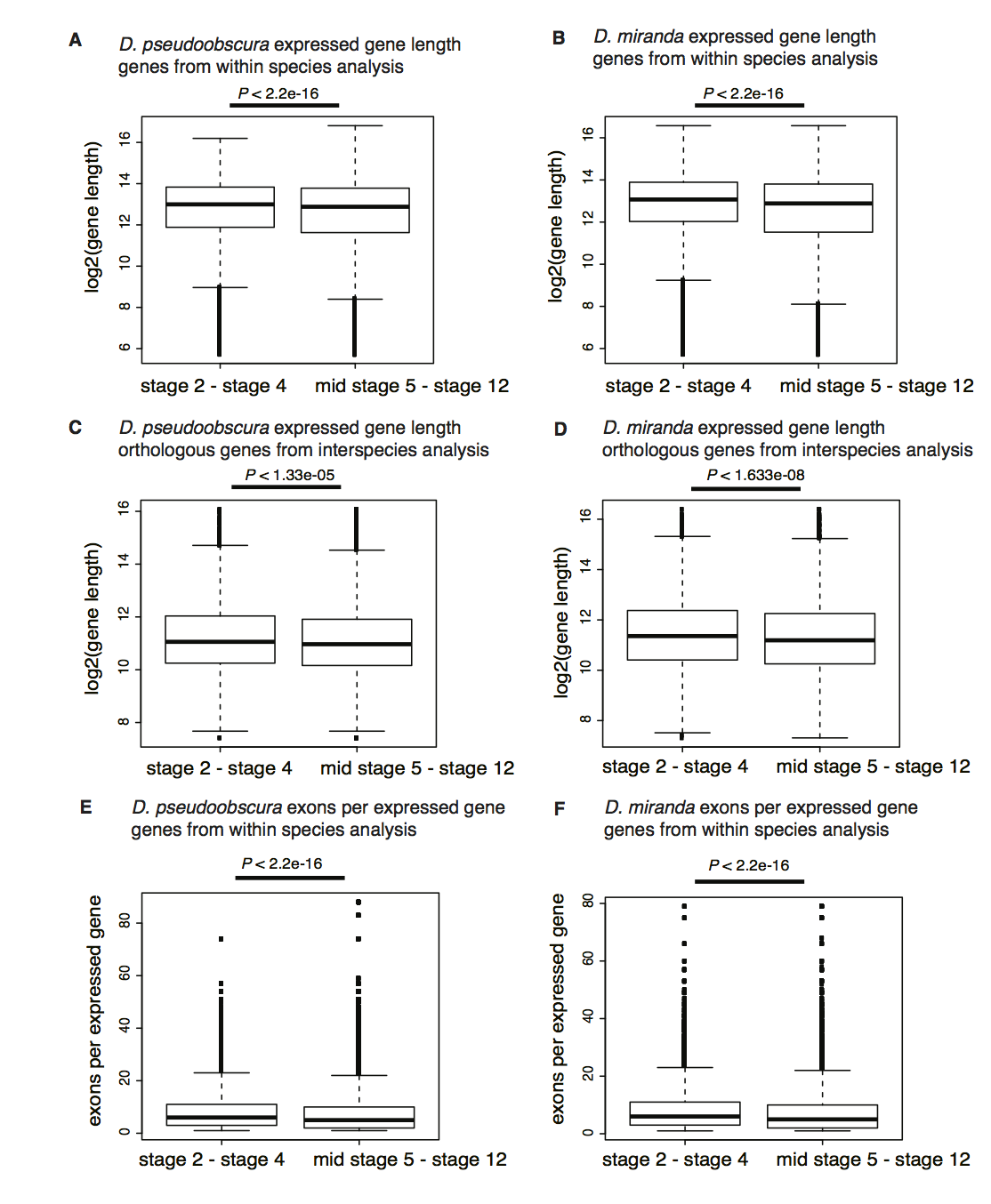

Supplement: S9 Fig — Log2(length) of genes expressed in early (stage 2 –stage 4) and later (mid stage 5 –stage 12) embryonic stages from within species analyses in A) D. pseudoobscura and B) D. miranda. Log2(length) of orthologous genes from interspecies analysis expressed in early (stage 2 –stage 4) and later (mid stage 5 –stage 12) embryonic stages in C) D. pseudoobscura and D) D. miranda. Number of exons per genes expressed in early (stage 2 –stage 4) and later (mid stage 5 –stage 12) embryonic stages from within species analyses in E) D. pseudoobscura and F) D. miranda. P-values indicate the results of Wilcoxon rank sum tests. (TIF) [file pgen.1006464.s009.tif]

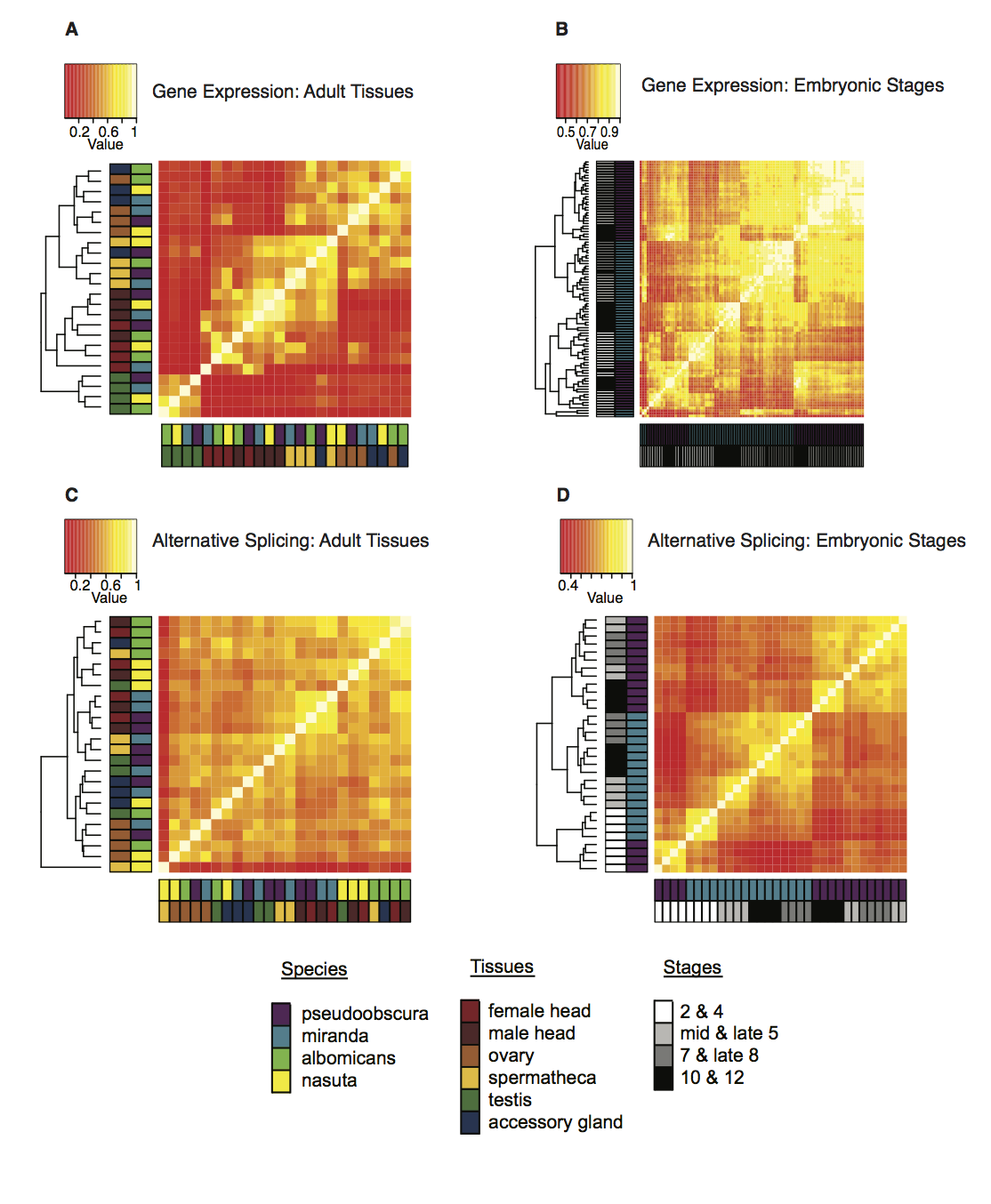

Supplement: S10 Fig — Pearson correlations based on gene expression (TPM) for genes orthologous in adult tissues (n = 3005) (A) and embryonic stages (n = 6707) (B). Pearson correlations based on alternative splicing (Ψ) for exons orthologous and annotated as alternatively spliced in at least one sample in adult tissues (n = 472) (C) and embryonic stages (n = 1122) (D). (TIF) [file pgen.1006464.s010.tif]

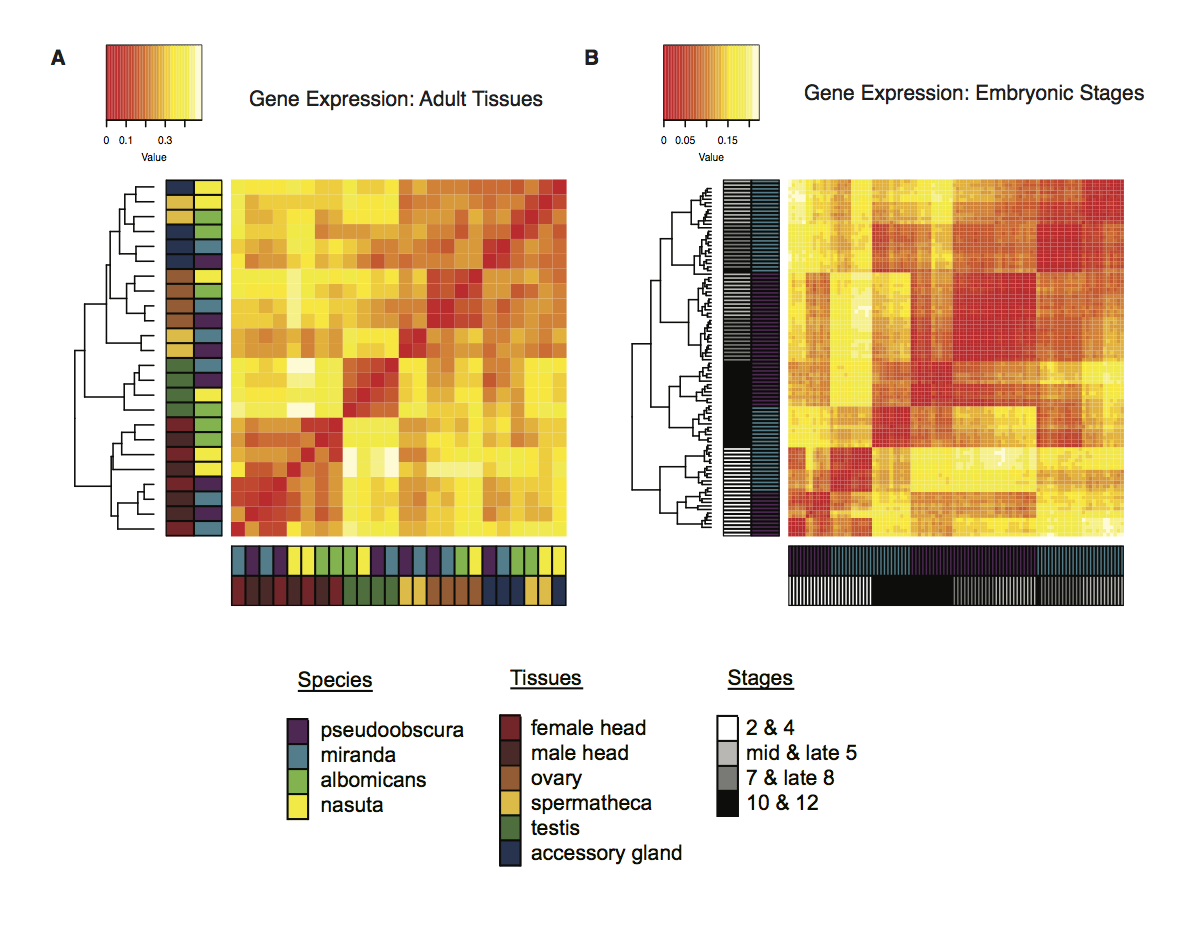

Supplement: S11 Fig — Heatmaps based on Jensen-Shannon divergence of gene expression for genes orthologous in adult tissues (n = 3005) (A) and embryonic stages (n = 6707) (B). (TIF) [file pgen.1006464.s011.tif]

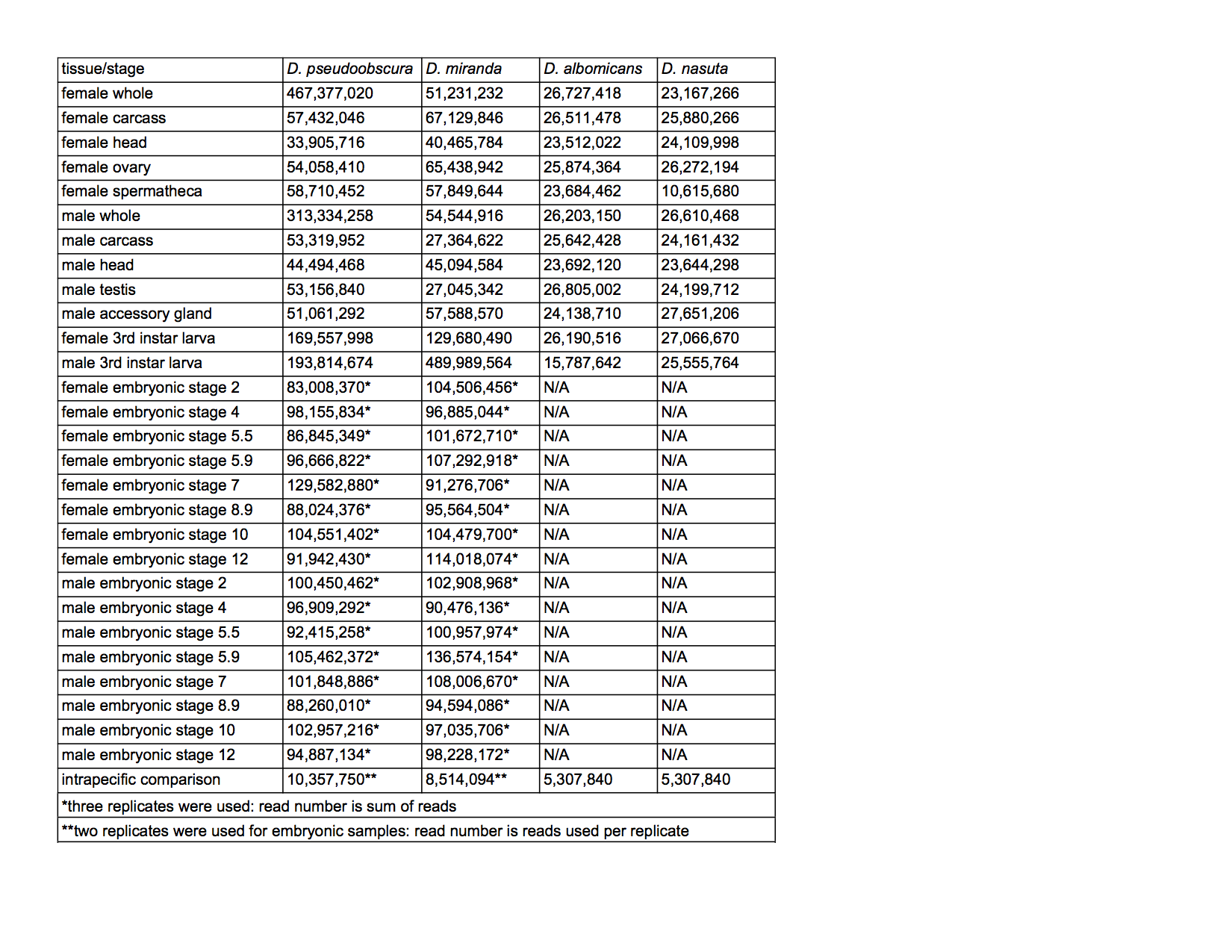

Supplement: S1 Table — The numbers of pairs of paired-end RNA-seq reads used for interspecies analyses, broken down by species and tissue/sex/stage, and intraspecies analysis. “5.5” = mid stage 5; “5.9” = late stage 5; “8.9” = late stage 8 (TIF) [file pgen.1006464.s012.tif]

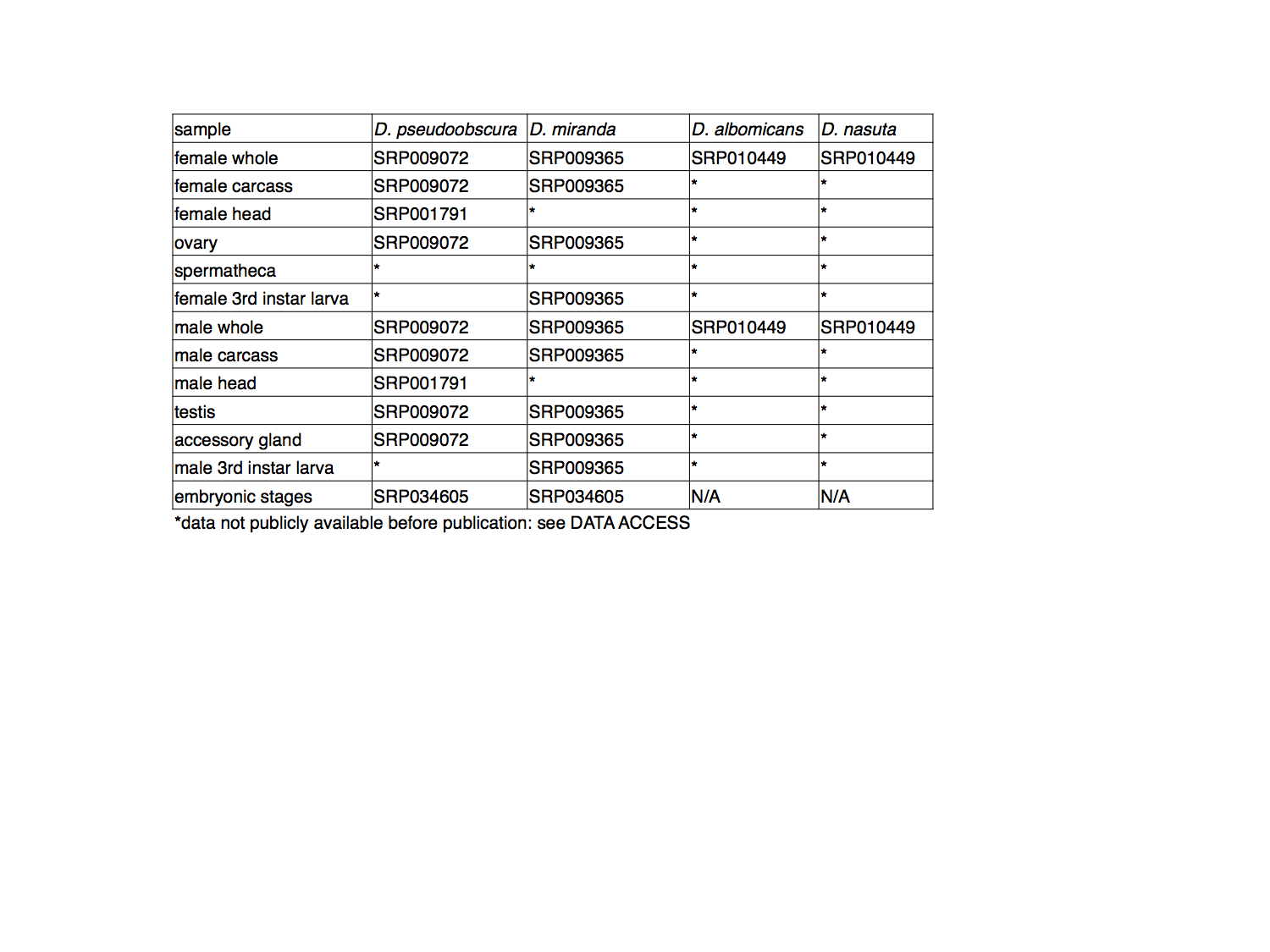

Supplement: S2 Table — (TIF) [file pgen.1006464.s013.tif]

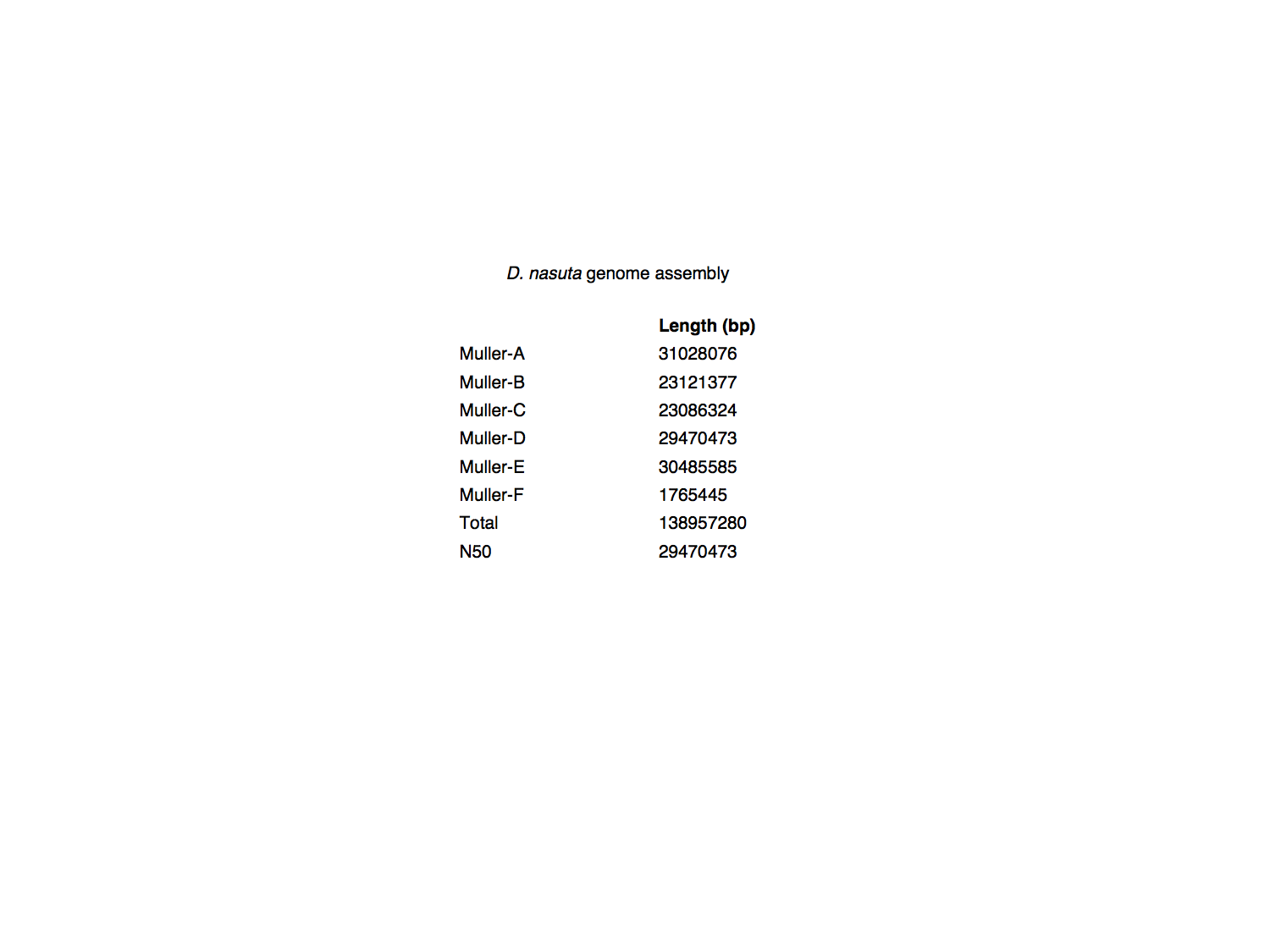

Supplement: S3 Table — (TIF) [file pgen.1006464.s014.tif]

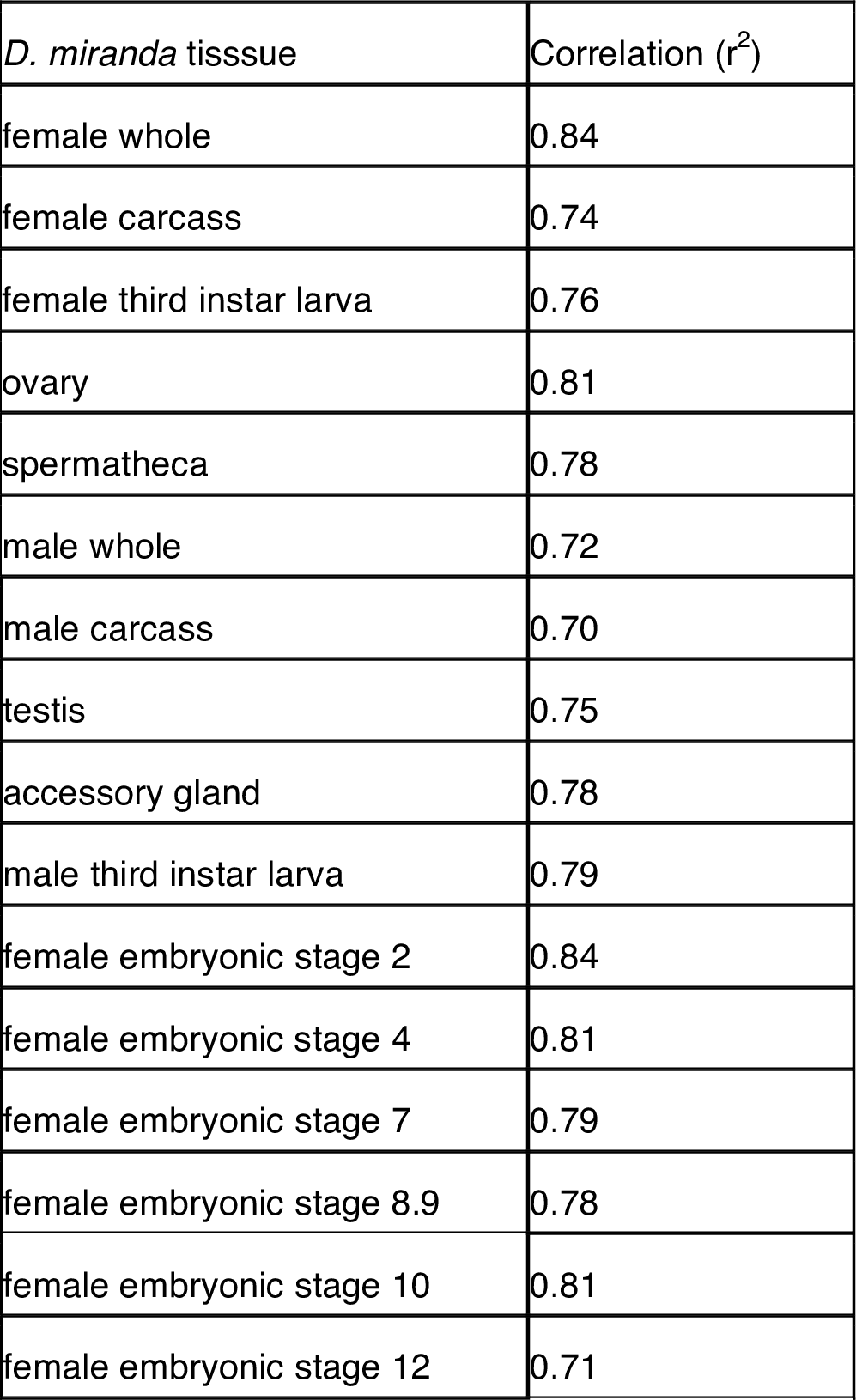

Supplement: S4 Table — The first pipeline, described in Materials and Methods, used MATS to annotate alternatively spliced exons and compute their Ψ values. The second pipeline used AltEventFinder to annotate alternatively spliced exons and MISO to compute their Ψ values. (TIF) [file pgen.1006464.s015.tif]
